# Hydrogen Sulfide Inhibits High Glucose-Induced Neuronal Senescence by Improving Autophagic Flux *via* Up-regulation of SIRT1

**DOI:** 10.3389/fnmol.2019.00194

**Published:** 2019-08-20

**Authors:** Lei Wu, Ying Chen, Chun-Yan Wang, Yi-Yun Tang, Hong-Lin Huang, Xuan Kang, Xiang Li, Yu-Rong Xie, Xiao-Qing Tang

**Affiliations:** ^1^Institute of Neuroscience, Hengyang Medical College, University of South China, Hengyang, China; ^2^Hunan Province Cooperative Innovation Center for Molecular Target New Drug Study, Institute of Pharmacy and Pharmacology, University of South China, Hengyang, China; ^3^Department of Pharmacology, The Central Hospital of Hengyang, Hengyang, China; ^4^Institute of Neurology, the First Affiliated Hospital, University of South China, Hengyang, China; ^5^College of Chemistry and Chemical Engineering, University of South China, Hengyang, China

**Keywords:** hydrogen sulfide, high glucose, SIRT1, autophagic flux, neuronal senescence

## Abstract

Hyperglycemia, a key characteristic and risk factor for diabetes mellitus (DM), causes neuronal senescence. Hydrogen sulfide (H_2_S) is a novel neuroprotectant. The present work was to investigate the potential effect of H_2_S on hyperglycemia-induced neuronal senescence and the underlying mechanisms. We found that NaHS, a donor of H_2_S, inhibited high glucose (HG)-induced cellular senescence in HT22 cells (an immortalized mouse hippocampal cell line), as evidenced by a decrease in the number of senescence associated-β-galactosidase (SA-β-gal) positive cells, increase in the growth of cells, and down-regulations of senescence mark proteins, p16^INK4a^ and p21^CIP1^. NaHS improved the autophagic flux, which is judged by a decrease in the amount of intracellular autophagosome as well as up-regulations of LC3II/I and P62 in HG-exposed HT22 cells. Furthermore, blocked autophagic flux by chloroquine (CQ) significantly abolished NaHS-exerted improvement in the autophagic flux and suppression in the cellular senescence of GH-exposed HT22 cells, which indicated that H_2_S antagonizes HG-induced neuronal senescence by promoting autophagic flux. We also found that NaHS up-regulated the expression of silent mating type information regulation 2 homolog 1 (SIRT1), an important anti-aging protein, in HG-exposed HT22 cells. Furthermore, inhibition of SIRT1 by sirtinol reversed the protection of H_2_S against HG-induced autophagic flux blockade and cellular senescence in HT22 cells. These data indicated that H_2_S protects HT22 cells against HG-induced neuronal senescence by improving autophagic flux *via* up-regulation of SIRT1, suggesting H_2_S as a potential treatment strategy for hyperglycemia-induced neuronal senescence and neurotoxicity.

## Introduction

Diabetes mellitus (DM) increases the risk of central nervous system disease leading to encephalopathy (Sima et al., 2004), because hyperglycemia, the mainly characterized of DM, triggers neuronal damage (Fan et al., 2016; Kumar et al., 2017). Increasing evidence demonstrated that hyperglycemia induces neuronal senescence (Reno et al., 2013; Kaur et al., 2017; Yerra et al., 2017; Zhang et al., 2019). Therefore, increasing the defenses against hyperglycemia-induced neuronal senescence represents a future strategy for diabetic encephalopathy. Hydrogen sulfide (H_2_S), the third gaseous signaling molecule (Wang, 2010), serves as an important neuroprotective agent in the central nervous system (Hu et al., 2009; Li et al., 2014; Wei et al., 2014). It has been confirmed that H_2_S protects neuronal cells against stress-induced senescence (Liu et al., 2013; Xie et al., 2014). Furthermore, H_2_S has emerged as an important therapeutic target for the treatment of neurological diseases (Suzuki et al., 2011; Si et al., 2013). Therefore, we speculated that H_2_S also protects neurons against hyperglycemia-induced cellular senescence.

Autophagic flux, which regulates cell survival, metabolism and senescence (Mizushima et al., 2008; Shen et al., 2015), includes the formation of autophagic bodies and autophagic lysosomes, and the degradation of autophagy lysosomal inclusions (Lim et al., 2014). Recent evidence suggests that blocked autophagic flux contributes to neurodegeneration while improving autophagic flux has antagonistic effects on neurodegeneration (Nixon, 2013; Menzies et al., 2015; Lumkwana et al., 2017). Moreover, it has been reported that autophagic flux impairment induced by hyperglycemia is an essential pathogenic factor contributing to several DM-associated complications (Xie et al., 2016; Li Y. et al., 2017). Therefore, we hypothesized that block of autophagic flux mediates high glucose (HG)-induced neuronal senescence. Notably, H_2_S plays an important role in regulating autophagic flux (Xie et al., 2015). Consequently, we speculated that the protection of H_2_S against HG-induced neuronal senescence is by improving autophagic flux.

Silent mating type information regulation 2 homolog 1 (SIRT1) is an NAD^+^-dependent deacetylase (Cohen et al., 2004; Bordone and Guarente, 2005). In the brain, SIRT1 is mainly expressed in neurons and has numerous biological functions, including regulation differentiation, metabolism, cell survival, and aging (Qadir and Anwar, 2017). It has been reported that SIRT1 activation attenuates hyperglycemia and plays an important mediatory role in treating diabetic complication (Li X. N. et al., 2017; Zhang Q. et al., 2018; Zhang Y. et al., 2018). Furthermore, SIRT1 improves autophagic flux (Zhang et al., 2016; Liu et al., 2017). Based on that H_2_S up-regulates the expression of SIRT1 in neuron (Li et al., 2014), we hypothesized that SIRT1 mediates the protection of H_2_S against HG-triggered neuronal senescence by improving autophagic flux.

The present work identified that H_2_S inhibits HG-induced cellular senescence in HT22 cells, an immortalized mouse hippocampal cell line (Liu et al., 2009), and that H_2_S significantly attenuated HG-induced autophagy flux obstruction in HT22 cells. Furthermore, Chloroquine (CQ), an autophagic flux inhibitor, reversed the inhibitory roles of H_2_S in HG-induced autophagic flux obstruction and cellular senescence in HT22 cells. We also found that H_2_S significantly up-regulated SIRT1 expression in HG-exposed HT22 cells. Furthermore, Sirtinol, a SIRT1 inhibitor, reversed the inhibitory roles of H_2_S in HG-induced autophagic flux obstruction and cellular senescence in HT22 cells. Taken together, we demonstrated the protection of H_2_S against HG-induced cellular senescence of HT22 cells, as a result of improvement of autophagic flux *via* up-regulation of SIRT1.

## Materials and Methods

### Materials

Glucose, Sodium hydrosulfide (NaHS, a donor of H_2_S), CQ (inhibitor of autophagic flux), 3-methyladenine (3-MA, inhibitor of autophagic flux), Sirtinol (the specific inhibitor of SIRT-1), and trypan blue were obtained from Sigma Chemical Company (St. Louis, MO, USA). Specific monoclonal antibodies p62 and LC3 were purchased from Cell Signaling Technology. Specific monoclonal anti-SIRT1 antibody was obtained from Abcam (Hong Kong, China). Specific antibodies of p16^INK4a^ and p21^CIP1^ were purchased from OriGene Technologies.

### Methods

#### Cell Cultures

The mouse hippocampal neuron HT22 cell line was obtained from China Center for Type Culture Collection (Wuhan, China) and was used for all experiments. The cells were grown in Dulbecco’s Modified Eagle’s Medium (DMEM), which contains 10% FBS, 100 IU/mL of penicillin and 100 mg /mL of streptomycin, at 37°C and 5% CO_2_, under humidified atmosphere. The culture medium was changed every 3 days to ensure stable nutritional level.

#### Senescence Associated-β-Galactosidase (SA-β-gal) Assay

HT22 cells were fixed with 4% paraformaldehyde in 0.1 M phosphate-buffered saline (PBS) for 10 min. After three washes with PBS, HT22 cells were dyed with senescence associated-β-galactosidase (SA-β-gal) incubation overnight at 37°C under dry environment. HT22 cells were observed under an optical microscope (CKX41 SF, OLYMPUS, Japan). Living cells displayed normal nuclear size and morphology, whereas senescent cells with the characteristic morphology (enlarged and flattened) and positivity or SA-β-gal staining (turquoise color). The number of SA-β-gal-positive cell was counted to determine the percentage of senescent cells.

#### HT22 Cells Growth Curve Draw by Trypan Blue Counting

HT22 cells in logarithmic growth phase were seeded in 24-well plate with 1 × 10^4^ cells at each well. After the density of HT22 cells at 80%, cells were exposed to different experimental treatment. Three holes were randomly sampled for trypan blue count every day and counted for 7 days. For trypan blue staining, cell suspension was incubated with trypan blue in equal volume for 2 min and counting the unstained living cells on hemocytometer. The growth curve of the cell was plotted with the mean value of cell density per day as the ordinate and the growing days as the abscissa.

#### Transmission Electron Microscope

HT22 cells in logarithmic phase growth were seeded in a culture dish and were exposed to different experimental treatment for 48 h. After the treatment, cells were washed twice with PBS and fixed with 2.5% glutaraldehyde solution for 30 min at 4°C. Cells were harvested in a 1.5 ml of EP tube with 2.5% glutaraldehyde solution and conserved at 4°C. Samples were tested in Shanghai Fu cheng biology co., Ltd. Briefly, cells were dehydrated, embedded, sliced into 60 nm sections and stained with uranyl acetate at room temperature for 15 min, followed by lead citrate at room temperature for 15 min. Autophagosome and autolysosome formation were observed by transmission electron microscope.

### Western Blots Analysis

HT22 cell lysates were used to detect the protein expressions of p16^INK4a^, p21^CIPL^, SIRT1, LC3, and p62. After the exposure was terminated, cells were washed with PBS and then lysed in an ice-cold lysis buffer (20 mM Tris-HCl, pH 7.5, 150 mM NaCl, 1% Triton X-100, 1 mM phenyl methyl sulphonyl fluoride (PMSF), 1 mM Na_3_VO_4_, leupeptin, and EDTA) for 30 min. Liquid supernatant was collected after centrifugation for 10 min at 5,000 *g*. The protein concentration was determined by BCA Protein Assay Kit (Beyotime, Shanghai, China). The samples were diluted with sample buffer (Beyotime, Shanghai, China) at 100°C for 5 min and were separated by 10% sodium dodecyl sulfate-polyacrylamide gel (SDS-PAGE). And then, the proteins were transferred electrophoretically to polyvinylidene fluoride (PVDF) membranes. Non-specific protein binding was incubated with 5% non-fat dried milk in TBST buffer (pH 7.6, 3.03 g Tris base, 18.8 g glycine, 1 g SDS, plus 1 ml Tween-20) for 2 h at room temperature. The membranes were incubated overnight at 4°C with diluted primary antibody (anti-p16^INK4a^, 1:2,000; anti-p21^CIPL^, 1:2,000; anti-SIRT1, 1:2,000; anti-p62, 1:1,000 and anti-LC3, 1:1,000). The membranes were then washed three times for 5 min each time with TBST, and incubated with HRP-conjugated secondary antibody (1:5,000, Protein Tech, SA00001-2) at room temperature for 2 h. Protein bands were analyzed using the enhanced chemiluminescence detection system (BeyoECL Plus kit, Beyotime, P0018). Integrated optical densities were analyzed by using ImageJ software.

### Statistical Analysis

Statistical analysis of all data was performed by SPSS 18.0 software. Data are displayed as the mean ± SEM. Umbers of the biological replicates (*n*) are noted in the figure legends. The significance of intergroup differences was evaluated by LSD-t to compare the data of different experimental groups with multiple comparisons. Differences were considered significant at *P* < 0.05.

## Results

### H_2_S Antagonizes HG-Induced Cellular Senescence in HT22 Cells

**Figure 1:**
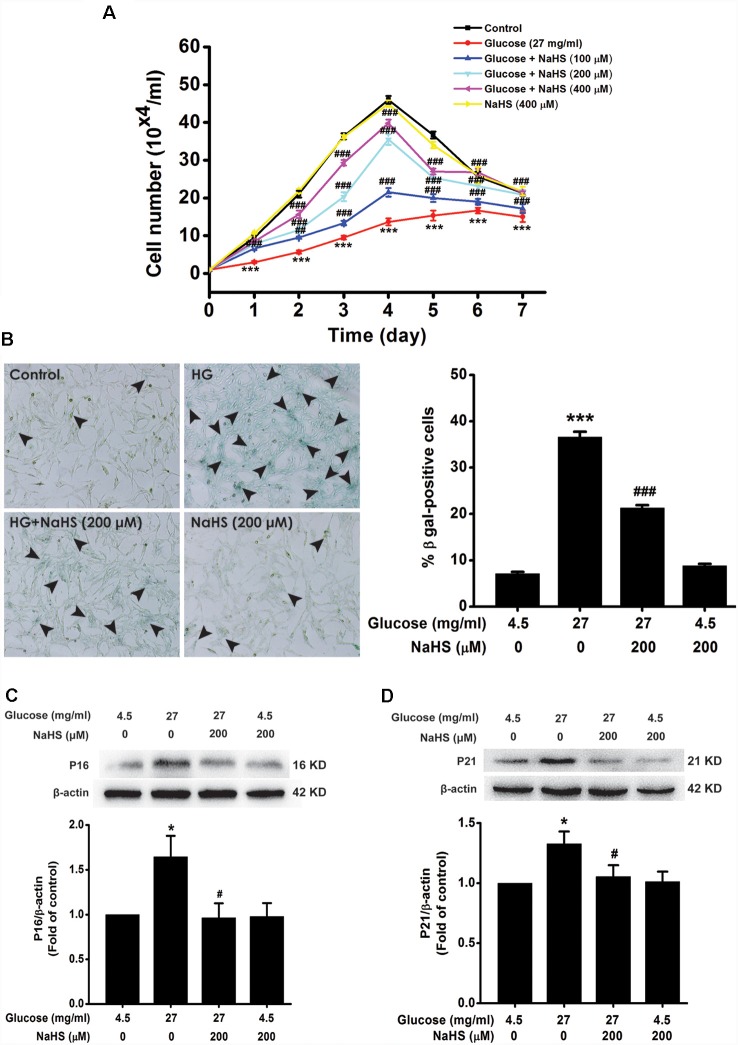
Effect of hydrogen sulfide (H_2_S) on high glucose (HG)-elicited cellular senescence in HT22 cells. **(A)** Cell growth curves were generated using Trypan blue stain assays. **(B)** Representative images of senescent cells that were stained using senescence associated-β-galactosidase (SA-β-gal; Left, Magnification ×100) and quantitative analysis of the SA-β-gal positive cells (Right). The black arrows indicate the senescent cells, **(C,D)** The expressions of p16^INK4a^ and p21^CIP1^ in HT22 cells were detected by Western blotting. Values were expressed as the mean ± SEM, *n* = 3. *P < 0.05, ***P < 0.001, vs. control group; ^#^*P* < 0.05, ^##^*P* < 0.01, ^###^*P* < 0.001 vs. HG-treated alone group.

We investigated the effect of H_2_S on HG-induced cellular senescence in HT22 cells. As illustrated in Figure 1A, the cell growth curves show that the growth of HT22 cell exposed by HG (27 mg/ml, for 48 h) was increased by pretreatment with 100, 200, or 400 μM of NaHS for 30 min (Day 1, *F*_(5,12)_= 39.718, *P* < 0.001; Day 2, *F*_(5,12)_= 83.543, *P* < 0.001; Day 3, *F*_(5,12)_ = 235.790, *P* < 0.001; Day 4, *F*_(5,12)_ = 149.725, *P* < 0.001; Day 5, *F*_(5,12)_= 65.619, *P* < 0.001; Day 6, *F*_(5,12)_ = 10.661, *P* < 0.001; Day 7, *F*_(5,12)_ = 6.735, *P* < 0.01), respectively. In addition, the number of SA-β-gal positive cell was increased in HT22 cells treated with 27 mg/ml of HG for 48 h (*F*_(3,16)_ = 393.481, *P* < 0.001); However, pretreated with NaHS (200 μM for 30 min) reduced the number of SA-β-gal positives cell in HT22 cells exposed to 27 mg/ml of HG for 48 h (*F*_(3,16)_ = 393.481, *P* < 0.001, Figure 1B). Furthermore, pretreatment of HT22 cells with 200 μM NaHS for 30 min significantly reversed the up-regulation of p16^INK4a^ (*F*_(3,8)_ = 4.257, *P* < 0.05; Figure 1C) and p21^CIP1^ (*F*_(3,8)_ = 3.559, *P* < 0.05; Figure 1D) protein expression induced by HG (27 mg/ml, for 48 h). Taken together, these data indicated that H_2_S inhibits HG-induced cellular senescence in HT22 cells.

### H_2_S Rescues HG-Impaired Autophagic Flux in HT22 Cells

**Figure 2:**
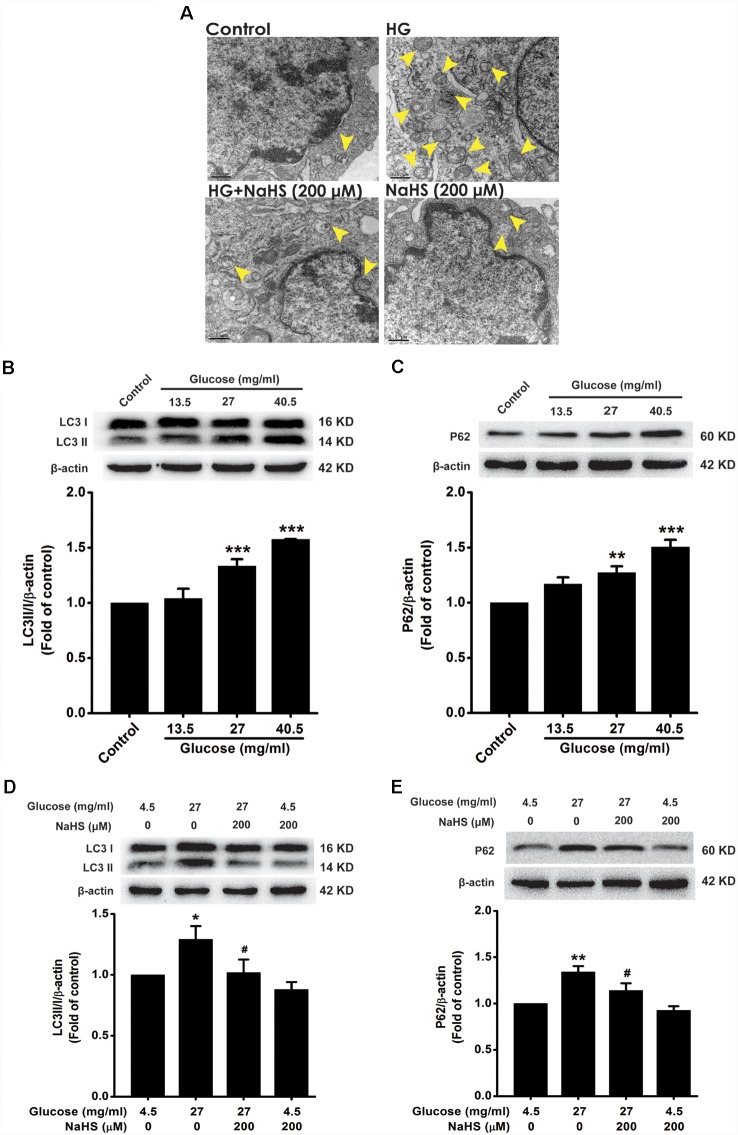
Effect of H_2_S on HG-elicited disruption of autophagic flux in HT22 cells. **(A)** Transmission electron microscope observed the number of autophagosome. The yellow arrows indicate autophagosome. **(B–E)** The expressions of LC3II/LC3I and P62 in HT22 cells were detected by Western blotting. Values were expressed as the mean ± SEM, *n* = 3. *P < 0.05, **P < 0.01, ***P < 0.001, vs. control group; ^#^*P* < 0.05, vs. HG-treated alone group.

To explore whether autophagic flux involves in the protection of H_2_S against HG-induced cellular senescence in HT22 cells, we investigated the effect of NaHS on the autophagic flux in HG-exposed HT22 cells. The number of autophagosomes in HT22 cells exposed to HG (40.5 mg/ml, for 48 h) was higher than that of the control group, which was suppressed by pretreatment with NaHS (200 μM, for 30 min; Figure 2A). In addition, after exposure to HG (27, 40.5 mg/ml) for 48 h, the expressions of LC3 II/I (*F*_(3,8)_ = 25.027, *P* < 0.001; Figure 2B) and P62 (*F*_(3,8)_ = 16.177, *P* < 0.01; Figure 2C) were significantly increased in HT22 cells. However, pretreatment with NaHS (200 μM, for 30 min) significantly down-regulated the expressions of LC3 II/I (*F*_(3,8)_ = 4.452, *P* < 0.05; Figure 2D) and P62 (*F*_(3,8)_ = 11.547, *P* < 0.05; Figure 2E) in HG-exposed HT22 cells. Collectively, these results demonstrated that H_2_S reverses HG-induced autophagic flux block in HT22 cells.

### Blockage of Autophagic Flux Reverses the Improving Effect of H_2_S on Autophagic Flux in HG-Exposed HT22 Cells

**Figure 3:**
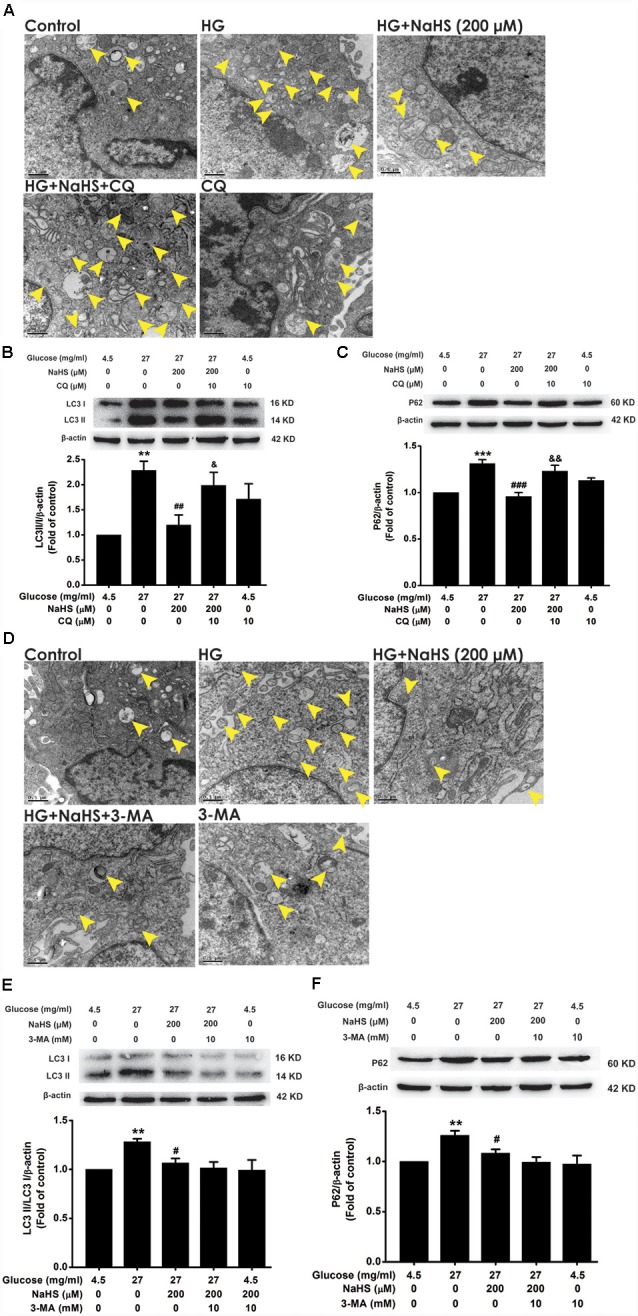
Effects of chloroquine (CQ) and 3-methyladenine (3-MA) on the improving role of H_2_S in autophagic flux in HG-exposed HT22 cells. **(A,D)** Transmission electron microscope images of HT22 cells showing the change in the number of autophagosome. The yellow arrows indicate autophagosome. **(B,C,E,F)** The expressions of LC3II/LC3I and P62 in HT22 cells were detected by Western blotting. Values were expressed as the mean ± SEM, *n* = 3. **P < 0.01, ***P < 0.001, vs. control group; ^#^*P* < 0.05, ^##^*P* < 0.01, ^###^*P* < 0.001, vs. HG-treated alone group; ^&^*P* < 0.05, ^&&^*P* < 0.01, vs. co-treated with NaHS and HG group.

3-MA and CQ are the specific autophagic flux inhibitors, which inhibit initial and late stage of autophagic flux (Caro et al., 1988; Ye et al., 2016), respectively. Next, we explored whether 3-MA and CQ abolish the improving effect of H_2_S on autophagic flux in HG-exposed HT22 cells. We found that CQ (10 μM) reversed the suppression of NaHS (200 μM) in HG (40.5 mg/ml, for 48 h)-induced increase in the number of autophagosome (Figure 3A) as well as upregulations of LC3II/LC3I (*F*_(4,10)_ = 6.151, *P* < 0.05; Figure 3B) and P62 (*F*_(4,10)_ = 13.498, *P* < 0.01; Figure 3C) in HT22 cells. However, 3-MA (10 mM) had no significant effect on the suppressive effect of NaHS (200 μM) on HG (40.5 mg/ml, for 48 h)-induced increase in the number of autophagosome (Figure 3D) as well as upregulations of LC3II/LC3I (*F*_(4,10)_ = 4.189, *P* > 0.05; Figure 3E) and P62 (*F*_(4,10)_ = 5.900, *P* > 0.05; Figure 3F) in HT22 cells. Taken together, these results indicated that H_2_S overcomes HG-induced disturbance in autophagic flux *via* restoring the late stage of autophagic flux.

### Blockage of Autophagic Flux by CQ Reverses the Protection of H_2_S Against HG-Induced Cellular Senescence in HT22 Cells

**Figure 4:**
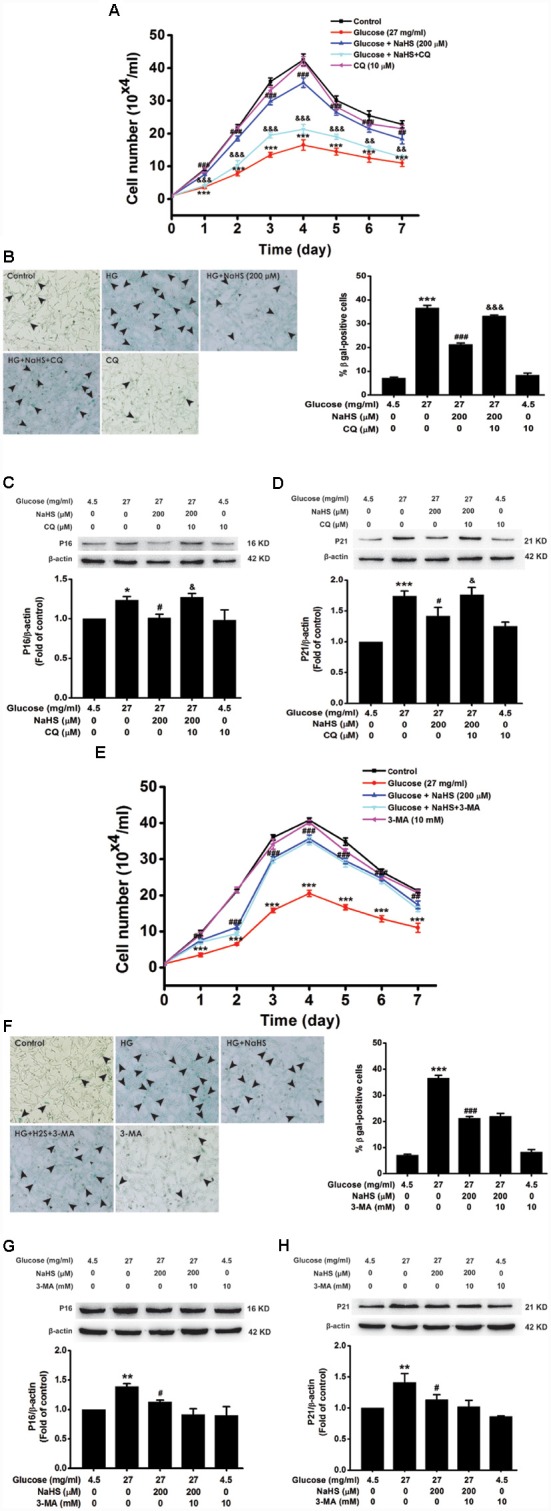
Effects of CQ and 3-MA on the protection of H_2_S against HG-induced cellular senescence in HT22 cells. **(A,E)** Cell growth curves were generated by using Trypan blue stain assays. **(B,F)** Representative images of senescent cells that were stained for SA-β-gal (Left, Magnification ×10) and quantitative analysis of the SA-β-gal positive cells (Right). The black arrows indicate the senescent cells, *n* = 5. **(C,D,G,H)** The expressions of p16^INK4a^ and p21^CIP1^ in HT22 cells were detected by Western blotting. Values were expressed as the mean ± SEM, *n* = 3. *P < 0.05, **P < 0.01, ***P < 0.001, vs. control group; ^#^*P* < 0.05, ^##^*P* < 0.01, ^###^*P* < 0.001 vs. HG-treated alone group. ^&^*P* < 0.05, ^&&^*P* < 0.01, ^&&&^*P* < 0.001, vs. co-treated with NaHS and HG group.

We further examined the effects of CQ and 3-MA on the protection of H_2_S against HG-induced cellular senescence in HT22 cells. Pretreatment with CQ (10 μM) reversed the suppression of NaHS (200 μM) in HG (40.5 mg/ml, for 48 h)-induced inhibition of the cell growth (Day 1, *F*_(4,10)_ = 32.703, *P* < 0.001; Day 2, *F*_(4,10)_ = 42.562, *P* < 0.001; Day 3, *F*_(4,10)_ = 94.889, *P* < 0.001; Day 4, *F*_(4,10)_= 58.601, *P* < 0.001; Day 5, *F*_(4,10)_ = 42.892, *P* < 0.001; Day 6, *F*_(4,10)_ = 26.198, *P* < 0.01; Day 7, *F*_(4,10)_ = 23.688, *P* < 0.01; Figure 4A), increase in the number of SA-β-gal positive cells (*F*_(4,20)_ = 330.457, *P* < 0.001; Figure 4B), as well as up-regulations of p16^INK4a^ (*F*_(4,10)_ = 4.161, *P* < 0.05; Figure 4C) and p21^CIP1^ (*F*_(4,10)_ = 11.886, *P* < 0.05; Figure 4D) in HT22 cells. However, pretreated HT22 cells with 3-MA (10 mM) had no significant effect on the suppressive effect of NaHS (200 μM) on HG (40.5 mg/ml, for 48 h)-induced inhibition of the cell growth (Day 1, *F*_(4,10)_ = 16.885, *P* > 0.05; Day 2, *F*_(4,10)_ = 170.863, *P* < 0.05; Day 3, *F*_(4,10)_ = 91.809, *P* > 0.05; Day 4, *F*_(4,10)_ = 94.120, *P* > 0.05; Day 5, *F*_(4,10)_ = 68.969, *P* > 0.05; Day 6, *F*_(4,10)_ = 44.611, *P* > 0.05; Day 7, *F*_(4,10)_ = 20.872, *P* > 0.05; Figure 4E), increase in the number of SA-β-gal positive cells (*F*_(4,20)_ = 185.297, *P* > 0.05; Figure 4F), as well as up-regulations of p16^INK4a^ (*F*_(4,10)_ = 4.841, *P* > 0.05; Figure 4G) and p21^CIP1^ (*F*_(4,10)_ = 5.801, *P* > 0.05; Figure 4H). These results indicated that improving autophagic flux contributes to H_2_S-exerted protection against HG-induced cellular senescence in HT22 cells.

### H_2_S Up-Regulates SIRT1 Expression in HG-Treated HT22 Cells

**Figure 5:**
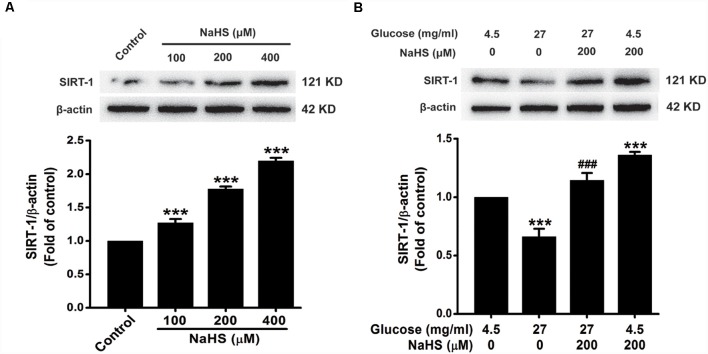
Effect of H_2_S on the expression of silent mating type information regulation 2 homolog 1 (SIRT1) in HT22 cells. **(A)** HT22 cells were treated with NaHS (100, 200 or 400 mM) for 24 h. **(B)** after pretreatment with NaHS (200 mM) for 30 min, HT22 cells were co-treated with HG (40.5 mg/ml) for 48 h. The expressions of SRT1 in HT22 cells were detected by Western blotting. Values were expressed as the mean ± SEM, *n* = 3. ***P < 0.001, vs. control group; ^###^*P* < 0.001 vs. HG-treated alone group.

In order to ascertain whether the protective effect of H_2_S is dependent on SIRT1, we first investigated the effect of H_2_S on the expression of SIRT1. As showed in Figure 5A, the expression of SIRT1 in HT22 cells were increased by treatment with NaHS (100, 200 or 400 μM) for 24 h (*F*_(3,8)_ = 169.526, *P* < 0.001). Furthermore, the down-regulation of SIRT1 caused by HG (40.5 mg/ml, for 48 h) was reversed by pretreatment with NaHS (200 μM, for 30 min; *F*_(3,8)_ = 37.762, *P* < 0.001; Figure 5B). These data indicated that H_2_S up-regulates the expression of SIRT1 in HT22 cells.

### Sirtinol, an Inhibitor of SIRT1, Reverses the Improving Effect of H_2_S on Autophagic Flux in HG-Exposed HT22 Cells

**Figure 6:**
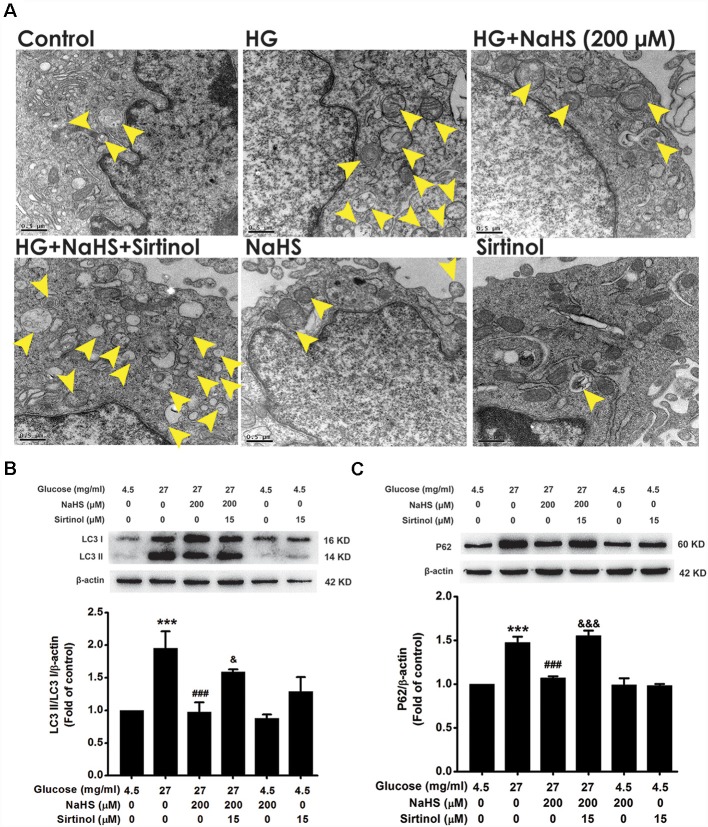
Effect of Sirtinol on the improving effect of H_2_S on autophagic flux in HG-exposed HT22 cells. **(A)** Representative transmission electron microscope images of HT22 cells showing the change in the number of autophagosome. The yellow arrows indicate autophagosome. **(B,C)** The expressions of LC3II/LC3I and P62 in HT22 cells were detected by Western blotting. Values were expressed as the mean ± SEM, *n* = 3. ***P < 0.001, vs. control group; ^###^*P* < 0.001, vs. HG-treated alone group. ^&^*P* < 0.05, ^&&&^*P* < 0.001, vs. co-treated with NaHS and HG group.

To confirm whether SIRT1 mediates the improving effect of H_2_S on autophagic flux in HG-exposed HT22 cells, we explored whether the SIRT1 inhibitor, Sirtinol, reverses this improving effect of H_2_S on autophagic flux. Pretreatment with Sirtinol (15 μM) abolished the suppression of NaHS (200 μM) in HG (40.5 mg/ml, for 48 h)-induced increase in the number of autophagosome (Figure 6A) as well as up-regulations of LC3II/LC3I (*F*_(5,12)_ = 7.733, *P* < 0.05; Figure 6B) and P62 protein (*F*_(5,12)_ = 29.863, *P* < 0.001; Figure 6C) in HT22 cells, which indicated that inhibited SIRT1 by Sirtinol reverses the improving effect of H_2_S on autophagic flux in HG-exposed HT22 cells.

### Sirtinol Abolishes the Protection of H_2_S Against HG-Induced Cellular Senescence in HT22 Cells

**Figure 7:**
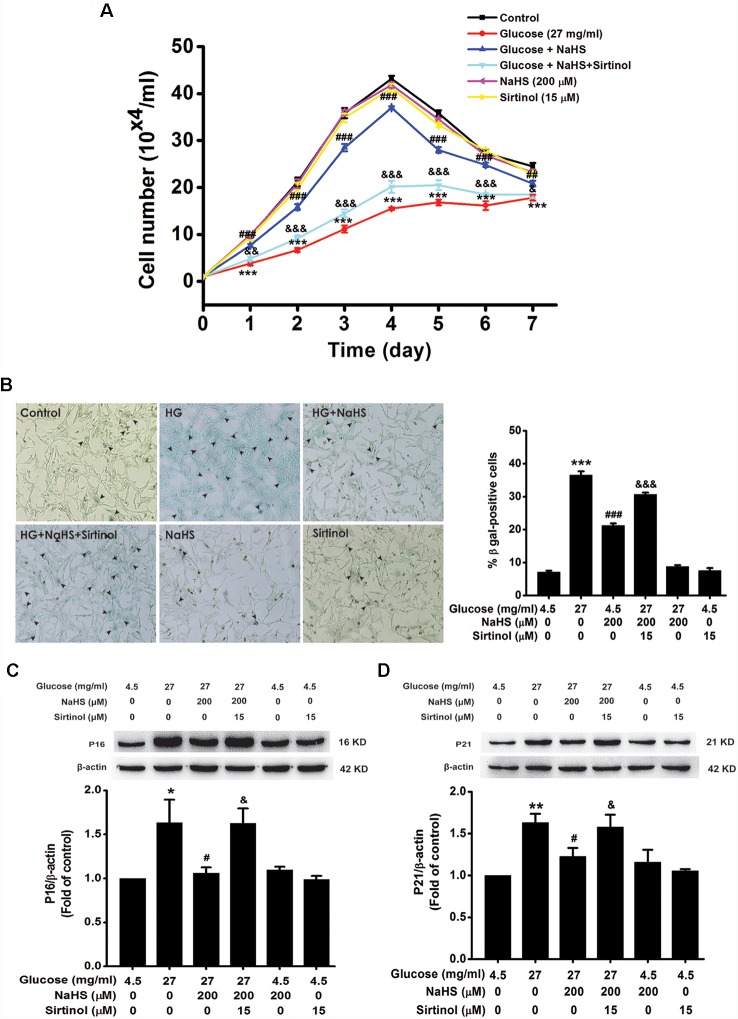
Sirtinol reversed H_2_S-inhibited cellular senescence in HG-exposed HT22 cells. **(A)** Cell growth curves were generated by using Trypan blue stain assays. **(B)** Representative images of senescent cells that were stained for SA-β-gal (Left, Magnification ×10) and quantitative analysis of the SA-β-gal positive cell (Right). The black arrows indicate the senescent cells. **(C,D)** The expressions of LC3II/LC3I and P62 in HT22 cells were detected by Western blotting. Values were expressed as the mean ± SEM, *n* = 3. *P < 0.05, **P < 0.01, ***P < 0.001, vs. control group; ^#^*P* < 0.05, ^##^*P* < 0.01, ^###^*P* < 0.001, vs. HG-treated alone group. ^&^*P* < 0.05, ^&&^*P* < 0.001, ^&&&^*P* < 0.001, vs. co-treated with NaHS and HG group.

Next, we explored whether inhibited SIRT1 abolishes the protection of H_2_S against HG-induced cellular senescence in HT22 cells. Pretreatment with Sirtinol (15 μM) reversed the suppression of NaHS (200 μM) in HG (40.5 mg/ml, for 48 h)-induced inhibition of the cell growth (Day 1, *F*_(5,12)_ = 29.984, *P* < 0.01; Day 2, *F*_(5,12)_ = 50.438, *P* < 0.001; Day 3, *F*_(5,12)_ = 181.255, *P* < 0.001; Day 4, *F*_(5,12)_ = 256.034, *P* < 0.001; Day 5, *F*_(5,12)_ = 155.338, *P* < 0.001; Day 6, *F*_(5,12)_ = 65.111, *P* < 0.001; Day 7, *F*_(5,12)_ = 16.613, *P* < 0.05; Figure 7A), increase in the number of SA-β-gal positive cells (*F*_(5,24)_ = 368.555, *P* < 0.001; Figure 7B), as well as upregulations of p16^INK4a^ (*F*_(5,12)_ = 5.609, *P* < 0.05; Figure 7C) and p21^CIP1^ (*F*_(5,12)_ = 6.522, *P* < 0.05; Figure 7D) in HT22 cells, which indicated that inhibited SIRT1 by sirtinol reverses the protection of H_2_S against HG-induced cellular senescence in HT22 cells.

## Discussion

Neuronal senescence plays key risk factors in hyperglycemia-induced neurotoxicity (Allen et al., 2005; Tomlinson and Gardiner, 2008). Our present work was to investigate whether H_2_S prevents hyperglycemia-induced neuronal senescence and the underlying mechanisms. The main findings of the present study are as followings: (1) NaHS (a donor of H_2_S) inhibits HG-induced cellular senescence of HT22 cells; (2) NaHS restores HG-induced autophagic flux dysfunction in HT22 cells; (3) inhibition of autophagic flux by CQ reversed H_2_S-restored autophagic flux in HG-exposed HT22 cells; (4) CQ abolishes the protective effects of H_2_S on HG-induced cellular senescence in HT22 cells; (5) H_2_S upregulates the expression of SIRT1 protein in HG-exposed HT22 cells; and (6) sirtinol, an inhibitor of SIRT-1, not only reverses the improvement of H_2_S in HG-induced autophagic flux disturbance in HT22 cells but also blocks the protection of H_2_S against HG-induced cellular senescence in HT22 cells. This is the first report that H_2_S rescues HG-induced neuronal senescence by improving autophagic flux through up-regulation of SIRT1.

Accumulating evidence demonstrates that long-term hyperglycemia causes neurotoxicity (Liu et al., 2014; Bahniwal et al., 2017; Li Y. et al., 2017; Renaud et al., 2018). It is well established that neuronal senescence plays a crucial role in the neurotoxicity of hyperglycemia (Sinadinos et al., 2014; Song et al., 2016; Zhu et al., 2018). Therefore, strategies to preserve neuronal cells by increasing the defenses of hyperglycemia-induced neuronal senescence are emerging as promising therapeutic approaches to prevent hyperglycemia-induced neurotoxicity. Due to their higher metabolic demands, HT22 cells require higher levels of glucose in normal growth media (25 mM glucose; Ward and Ergul, 2016). We have previously confirmed that treatment of HT22 cells with HG (150 mM) for 48 h, the cell viability was decreased to 70% compared with the control group (Zhu et al., 2018). In order to eliminate the effect of HG concentration on alteration of osmolarity, we have previously found that equal concentration of mannitol (150 mM) had no significant effect on the cell viability compared with the control group (Zhu et al., 2018). Similarly, Song et al. (2016) used 200 mM glucose to explore the effect of HG on senescence of neuronal cells. Therefore, in the present work, HT22 cells were exposed by 13.5–40.5 mg/ml of glucose to effectuate hyperglycemia-induced neuronal senescence *in vitro*. H_2_S, as a neuroprotector, has universal anti-senescence effects (Qi et al., 2012; Yang et al., 2013; Panthi et al., 2016), identifying as a potent preventive and therapeutic agent for senescence-associated disease (Zhang et al., 2013). Therefore, we explored whether H_2_S prevents hyperglycemia-induced neuronal senescence. The SA-β-gal (Dimri et al., 1995; Debacq-Chainiaux et al., 2009) and trypan blue (Tennant, 1964) are main staining methods for identifying senescence cell. The p16^INK4a^ (Baker et al., 2011) and p21^CIP1^ (Carreira et al., 2005) are the main cellular senescence biomarker protein. We examined these senescent makers to explore whether pretreatment with NaHS prevents HG-induced senescence in HT22 cells. Our results showed that NaHS decreased the number of SA-β-gal positive cells, increased the growth of cells, and down-regulated the expressions of senescence marker, p16^INK4a^ and p21^CIP1^, in HG-exposed HT22 cells. Our results indicated that H_2_S antagonizes HG-induced cellular senescence in HT22 cells. It has been reported that H_2_S reverses H_2_O_2_-induced senescence in SH-SY5Y cells (Xie et al., 2014). Thus, it is reasonable to believe H_2_S as a potentially promising therapeutic approach to the treatment of HG-induced neuronal senescence.

Next, we explored the mechanisms underlying this inhibitory role of H_2_S on HG-induced cellular senescence. Emerging evidence suggests that blockage of autophagic flux results in the decrease in degradation of misfolded proteins or damaged organelles, which in turn leads to cellular senescence (Lin et al., 2015; Han et al., 2016; Yin et al., 2017). In the present study, we found that accumulation of autophagosomes, increase in LC3II/I, and upregulation of P62 were displayed in HG-exposed HT22 cell. P62 connects between LC3 and ubiquitination substrate, which is degraded in autolysosomes (Bjørkøy et al., 2005). The aggregation of P62 is associated with autophagic degradation dysfunction (Komatsu and Ichimura, 2010). Thus, our results indicated that HG induces blockage of autophagic flux in HT22 cells. There is evidence that HG exposure reduces autophagic flux in cardiomyocyte (Hou et al., 2018). Therefore, we suggested that blockage of autophagic flux plays an important role in HG-induced senescence of HT22 cells. It has been reported that activating autophagic flux by H_2_S prevents HG-induced injury in H9C2 cells (Yang et al., 2017). Moreover, H_2_S-mediated autophagic flux reduces serum triglyceride in rat liver (Sun et al., 2015). Therefore, we investigated whether improving autophagic flux plays a key role in the protection of H_2_S against HG-induced cellular senescence in HT22 cells. Notably, NaHS reduced the number of autophagosomes, decreased the ratio of LC3II/I, and downregulated the expression of P62 in HG-exposed HT22 cell, which indicated the improving role of H_2_S in autophagic flux. To further validate whether improving autophagic flux mediates the protection of H_2_S against HG-induced cellular senescence, we explored whether the inhibition of autophagic flux abolishes the protection of H_2_S. Thus, two autophagy inhibitors, CQ and 3-MA, were used in our research. We found that blockage of autophagic flux by CQ reverses the protection of H_2_S against HG-induced cellular senescence in HT22 cells. However, 3-MA could not reverse the above effects. Consistent with this notion, prolonged treatment with 3-MA promotes autophagic flux under nutrient-rich conditions (Wu et al., 2010). CQ impairs lysosome function that restrains fusion of autophagosome and lysosome, while 3-MA is a type III phosphatidylinositol 3-kinase (PI3KCIII) inhibitor that inhibits autophagy from the initiation step (Pliyev and Menshikov, 2012). These results thus indicated that H_2_S may block lysosome to rescue HG-impaired autophagic flux in HT22 cells. Taken together, our outcomes indicated that improving autophagic flux mediates the antagonistic action of H_2_S in HG-induced cellular senescence in HT22 cells. It has been demonstrated that blocking autophagic flux reverses the protective effect of H_2_S against acrylonitrile-induced neurotoxicity (Yang et al., 2018). Therefore, we suggest that improving autophagic flux contributes to the protective effect of H_2_S against HG-induced neuronal senescence.

SIRT1 is a NAD^+^-dependent deacetylase. Increasing evidence confirms the anti-senescence effect of SIRT1 (Hwang et al., 2013; Herskovits and Guarente, 2014). While, SIRT1 deficiency induces cellular senescence (Furukawa et al., 2007). Furthermore, it has been reported that SIRT1 prevents oxidative stress-induced cellular senescence *via* restoration of autophagic flux (Han et al., 2016) and that up-regulation of SIRT1 by resveratrol enhances autophagic flux in human umbilical vein endothelial cell (Zhang et al., 2016). It is reasonable to believe that SIRT1 suppresses cellular senescence *via* improving autophagic flux. Does SIRT1 mediate the protection of H_2_S against HG-induced neuronal senescence in HT22 cells? It has been documented that SIRT1 mediates the roles of H_2_S in attenuating chronic restraint stress-induced cognitive impairment (Li X. N. et al., 2017) and inhibiting homocysteine-induced endoplasmic reticulum stress in PC12 cells (Wang et al., 2017). Therefore, it seems advisable to conjecture that SIRT1 mediates the protection of H_2_S against HG-triggered neuronal senescence. In the present work, we found that H_2_S up-regulated the expression of SIRT1 in HG-exposed HT22 cells and that Sirtinol, an inhibitor of SIRT1 abolished the protective effect of H_2_S on HG-induced cellular senescence in HT22 cells. These results indicated that SIRT1 mediates the protection of H_2_S against HG-triggered neuronal senescence in HT22 cell. This finding is consistent with the previous report that stimulating of SIRT1 by H_2_S antagonizes senescence in human fibroblasts (Sanokawa-Akakura et al., 2016) and H_2_O_2_-induced senescence in human umbilical vein endothelial cells (Suo et al., 2013). Furthermore, we found that inhibition of SIRT1 by sirtinol reversed the improving role of H_2_S in the autophagic flux of HG-exposed HT22 cells. Taken together, we suggested that SIRT1 mediates the protective effect of H_2_S against HG-induced senescence in HT22 cells *via* improving autophagic flux.

In summary, our research indicated that H_2_S increases autophagic flux by up-regulation of SIRT1 expression and thereby antagonizes HG-induced cellular senescence in HT22 cells. These findings provide a deeper mechanism explanation for the protection of H_2_S against hyperglycemia-induced neuronal senescence and suggest that H_2_S may be a prospective strategy for prevention of hyperglycemia-induced neurotoxicity.

## Data Availability

The raw data supporting the conclusions of this manuscript will be made available by the authors, without undue reservation, to any qualified researcher.

## Author Contributions

X-QT and H-LH conceived and designed the experiments. YC, LW, C-YW, Y-YT, XL, XK, and Y-RX performed the experiments. X-QT, YC, and LW analyzed the data. C-YW, Y-YT, XL, XK, and Y-RX contributed reagents, materials and analysis tools. X-QT and LW wrote and revised the manuscript.

## Conflict of Interest Statement

The authors declare that the research was conducted in the absence of any commercial or financial relationships that could be construed as a potential conflict of interest.
